# The SpineBox: A Freely Available, Open-access, 3D-printed Simulator Design for Lumbar Pedicle Screw Placement

**DOI:** 10.7759/cureus.7738

**Published:** 2020-04-20

**Authors:** William Clifton, Aaron Damon, Fidel Valero-Moreno, Eric Nottmeier, Mark Pichelmann

**Affiliations:** 1 Neurological Surgery, Mayo Clinic, Jacksonville, USA

**Keywords:** spine models, covid-19, simulation, medical education, spine surgery, lumbar spine, 3d printing, three-dimensional (3d) printing, neurosurgery, vertebrae

## Abstract

Background

The recent COVID-19 pandemic has demonstrated the need for innovation in cost-effective and easily produced surgical simulations for trainee education that are not limited by physical confines of location. This can be accomplished with the use of desktop three-dimensional (3D) printing technology. This study describes the creation of a low-cost and open-access simulation for anatomical learning and pedicle screw placement in the lumbar spine, which is termed the SpineBox.

Materials and methods

An anonymized CT scan of the lumbar spine was obtained and converted into 3D software files of the L1-L5 vertebral bodies. A computer-assisted design (CAD) software was used to assemble the vertebral models into a simulator unit in anatomical order to produce an easily prototyped simulator. The printed simulator was layered with foam in order to replicate soft tissue structures. The models were instrumented with pedicle screws using standard operative technique and examined under fluoroscopy.

Results

Ten SpineBoxes were created using a single desktop 3D printer, with accurate replication of the cortico-cancellous interface using previously validated techniques. The models were able to be instrumented with pedicle screws successfully and demonstrated quality representation of bony structures under fluoroscopy. The total cost of model production was under $10.

Conclusion

The SpineBox represents the first open-access simulator for the instruction of spinal anatomy and pedicle screw placement. This study aims to provide institutions across the world with an economical and feasible means of spine surgical simulation for neurosurgical trainees and to encourage other rapid prototyping laboratories to investigate innovative means of creating educational surgical platforms in the modern era.

## Introduction

Simulation in neurosurgical training is becoming a prominent adjunct to classical intraoperative teaching of surgical skills and classroom didactics for anatomical education [1,2]. The implementation of duty hour requirements and other tools to decrease fatigue and resident burn-out have presented a challenge to institutions in providing both adequate quantity of and quality surgical procedural experience in order to produce well-trained surgical physicians [3]. In the field of neurosurgery specifically, the rarity of cases and relatively low general incidence of neurosurgical diseases compared to other surgical subspecialties presents additional challenges to ensure adequate trainee exposure to surgical pathology [4]. Three-dimensional printing (3DP) has provided a means for recreating key points of anatomy for anatomical and procedural learning, especially in the realm of spinal surgery [5]. The feasibility of creating low-cost but high-quality 3DP simulation has been investigated, with good results [6-8]. As the number of desktop 3DP continues to move into a standard workflow for many institutions, there remains a large shortage of simulator designs that do not require purchase or extensive assembly [9].

In the current wake of the COVID-19 outbreak, the fragility of the worldwide educational platform in surgical training has been made apparent, and many trainees are unable to continue to practice surgical skills due to the massive decrease in elective case volumes and currently unspecified quarantine time [10]. Currently, the mainstay of surgical education is live operative experience, which has continued to remain unchanged for over a century. The recent world events have demonstrated the immediate need for research and innovation in producing broadly accessible simulators in order to maintain educational activities for surgical residents. Simulator designs for neurosurgical resident education and skills training can be developed and implemented using desktop 3D printing methods that are inexpensive and widely available [11-13]. This report describes a novel simulator design for spine anatomy and instruction of pedicle screw placement in the lumbar spine and provides a link to the Standard Tessellation Language (STL) file for immediate printing and use. This simulator can be used as an in-house or “take-home” method of instructing residents in lumbar pedicle screw placement.

## Materials and methods

Before the simulator was constructed, the desired primary learning goals of the simulator design were clearly established. This methodology has been shown to improve the teaching elements of simulators by directing learning objects to specific task-training [14,15]. The primary learning goal of the simulator was to instruct junior neurosurgical trainees in the anatomy and technique for pedicle screw placement in the lumbar spine. In order for this to be accomplished, the anatomical and physiologic components of the lumbar vertebrae needed to be replicated, apart from the creation of a simulated operative environment. The key components of the simulation that were included in the initial design planning were as follows: 1. representative surface anatomy of the L1-L5 vertebrae; 2. replication of the cortico-cancellous interface; 3. preservation of the natural anatomical location and direction of the lumbar pedicles at each level (e.g., lordosis); and 4. replication of an intraoperative environment with the operative field deep to a set surface level (i.e., located deep to a “skin-level” surface).

In order to produce lumbar vertebral models that adequately replicated normal surface anatomy and ultrastructure, an anonymized CT scan of the lumbar spine in an adult patient was acquired through formal IRB approval. The scan was inspected for any previous surgical defects or degenerative pathology that would interfere with the learning objectives for the simulation. The CT scan was uploaded into 3D Slicer (Slicer, v.4.0), an open-access DICOM (Digital Information and Communication in Medicine) viewing platform that allows for the creation of 3D anatomical models for 3DP and viewing. The L1-L5 vertebrae were individually selected using the threshold module, and the facet and intervertebral spaces were manually separated to produce individually segmented models of the lumbar vertebrae. This was accomplished by setting the threshold limits to 193-3,000 HU. Each lumbar vertebral model was then converted into an STL file. All five lumbar STL files were then uploaded into Meshmixer 2017 (Autodesk, San Rafael, CA), an open-access computer-assisted design (CAD) software platform that allows for manipulation and editing of STL files in a virtual space (Video 1). The L1-L5 STL files were inspected for the preservation of the anatomical elements allowing for proper identification of the pedicle (e.g., transverse process, mammillary process, pars interarticularis). Additionally, each model was edited in order to remove unnecessary infill elements that would consume unnecessary material and printing time, and also to prevent the creation of a hollow model for programming infill for replication of the cortico-cancellous interface. The lumbar vertebrae were then arranged in anatomical order, with preservation of the lumbar lordosis [16].

**Video 1:** Design and use of the SpineBox simulator

Using the Meshmixer CAD software, a virtual housing box was constructed in order to provide an operative field for the vertebral models. The X-Y dimensions of the box were adjusted to provide adequate space superiorly to the L1 vertebra and inferiorly to the L5 vertebra, and medial/lateral to the transverse processes at each level. The Z dimension was adjusted in order to provide coverage of the superior level over the spinous processes, thus creating a “work-in-a-hole” design for the finished simulation. The individual STL files were combined and saved as one object. The finished SpineBox STL file was then uploaded into ideaMaker (Raise3D Technologies, Irvine, CA), our laboratory’s chosen software for slicing and G-code preparation. At this point in the design, the L1-L5 vertebral models were “floating” within the simulator box and thus would not be able to be printed. Using the placement of support material within the slicing software, the L1-L5 vertebral models were connected to the floor of the simulator box, thus ensuring the ability to print the entire object in one session and minimizing assembly post-production. The sliced SpineBox STL file was converted into a G-code and printed using acrylonitrile butadiene styrene (ABS) on a Raise3D Pro Plus fused deposition modeling (FDM) 3D printer (Raise3D Technologies, Irvine, CA).

## Results

**Figure 1:**
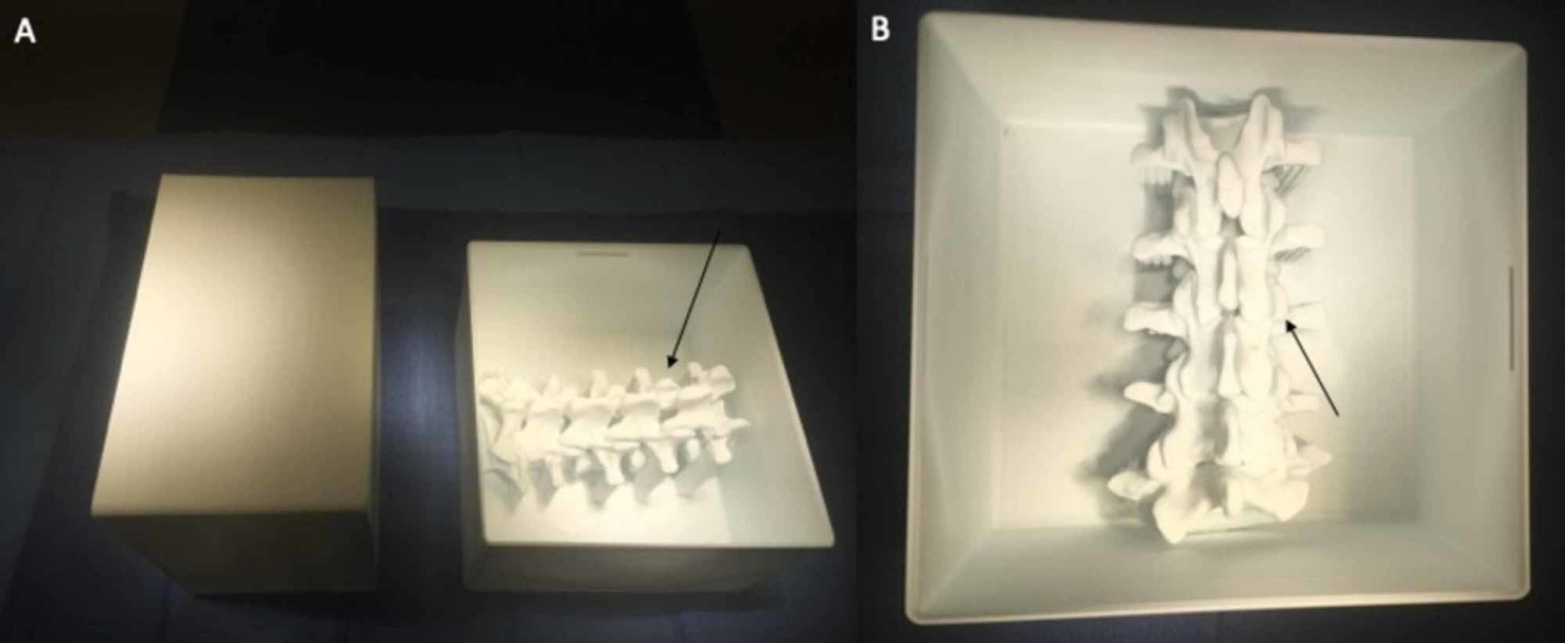
SpineBox finished print A: the lumbar vertebral models are located beneath the surface of the top of the housing unit (arrow); B: the posterior element landmarks for pedicle screw placement can be visualized from a posterior approach in a simulated operative field (arrow)

The SpineBox was successfully printed using 1.75 ±0.05-mm ABS filament through a 0.4-mm nozzle, bed temperature of 110 °C, nozzle temperature of 265 °C, and a print speed of 55 mm/second. The total print time was 30 hours and 17 minutes. The total material expenditure was 322 grams, and the total cost of each SpineBox was $9.68 ($0.03 per gram of ABS). Ten SpineBoxes were printed successfully in the initial pilot study. The dimensions of the finished print were 17.6 x 18.6 x 11.3 cm (Figure 1).

**Figure 2:**
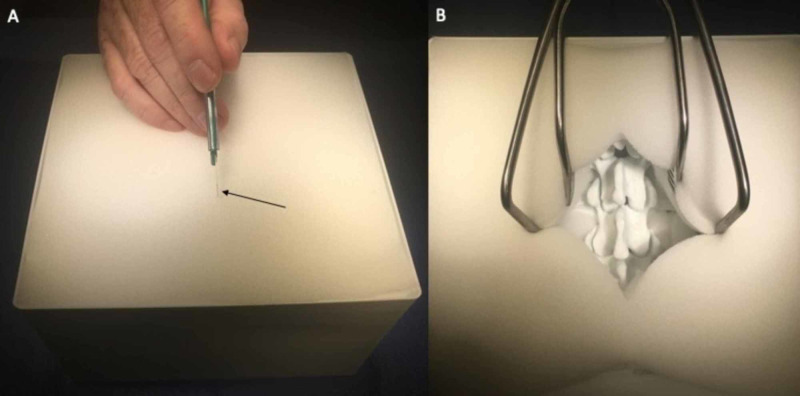
The use of polyurethane foam for soft tissue simulation A: soft polyurethane foam is used in layers to replicate visualization hindrance of the bony posterior elements of the lumbar spine, and can be cut with a scalpel (arrow); B: retractors can be used for exposure of the lumbar spine with similar instrument-working depth, as in a live operative scenario

In order to replicate soft tissue structures and create a hindrance of visualization during screw placement for increased educational value, flexible upholstery polyurethane foam sheets were acquired and cut to fit the X and Y dimensions of the simulator housing box (17.6 x 18.6 cm) and layered until the foam was flush with the top limit of the box. Smaller pieces were also cut and placed within the depths of the box underneath the transverse processes of the lumbar vertebrae. After complete assembly, the foam was able to be cut with a scalpel, and retractors could be placed in order to simulate the displacement of soft tissue for operative cavity visualization (Figure 2). The total cost of upholstery foam was $0.001 per cm^3^.

**Figure 3:**
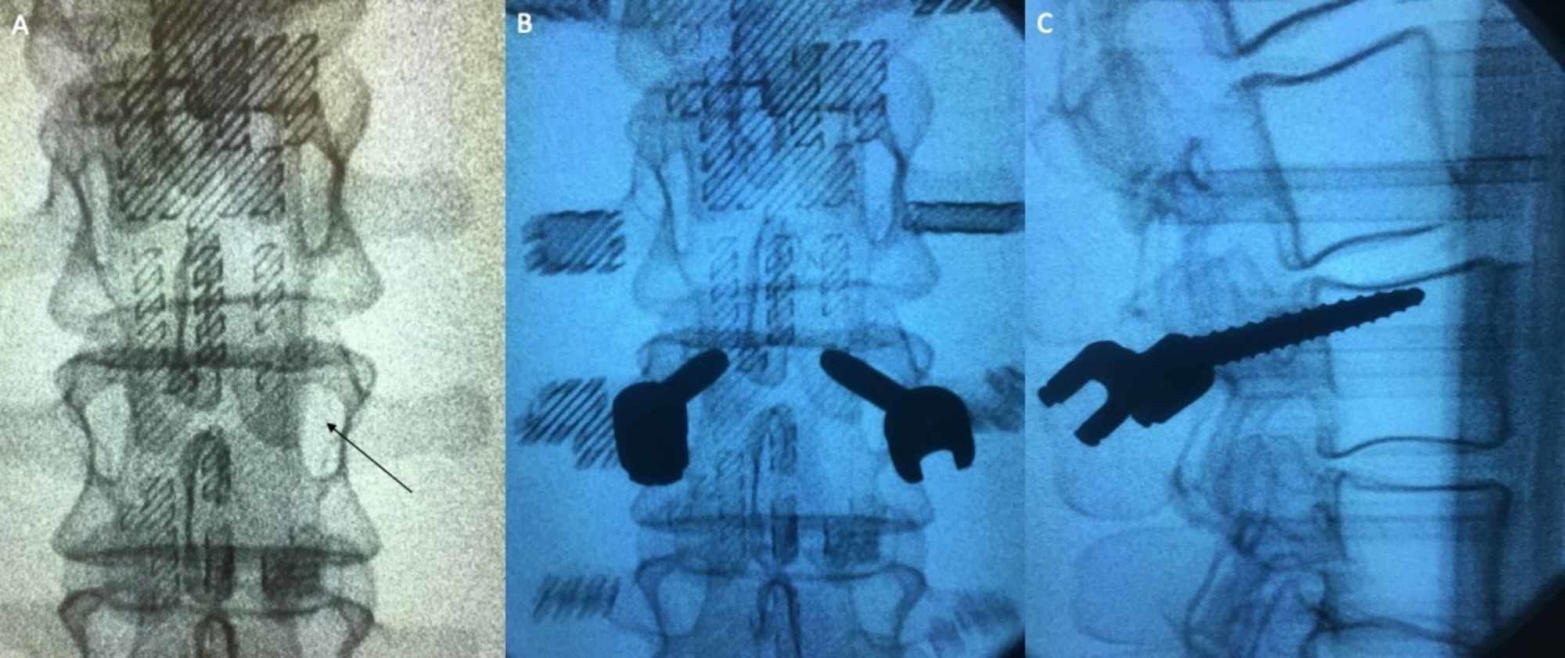
Fluoroscopic investigation of the SpineBox A: the pedicles (arrow) of the 3D printed lumbar vertebrae can be located on fluoroscopic imaging; B and C: pedicle screws (black objects) can be seen within the model after being placed, and graded based on location for both teaching and objective skill assessment

A total of 100 pedicle screws in 50 lumbar levels were placed within the 10 models. Five models were filled with polyisocyanate foam in order to replicate a previously validated method of simulating cancellous bone [17]. The other five models were sliced with a 25% infill in a honeycombed pattern within the vertebrae, which is another validated method of simulating vertebral cancellous bone [18]. Fluoroscopy was used to determine the ability of the model to demonstrate pedicle screw placement analogous to an intraoperative scenario. All pedicle screws within the models were able to be visualized with fluoroscopic localization (Figure 3). After the pedicle screw placement, the lumbar vertebral models were able to be removed from the housing unit and directly examined for breaches, enhancing the learning capabilities of the simulation.

## Discussion

Spine surgical simulation has made tremendous growth and improvements in quality over the last decade and the presence of elaborate simulators in the current field has shown the ability of 3DP to create new ways of simulating spine surgery for trainees [19,20]. Still, the ability of training institutions to acquire such simulation without purchase or considerable assembly requirements remains limited [21,22]. In the current global scenario, it has become increasingly important to have access to learning tools that are mobile and independent of hospital-related conditions. The recent outbreak of COVID-19 has diminished many neurosurgical learning opportunities throughout the world due to conference cancellations, decreased operating room utility, and quarantine policies [23]. In the present landscape of spine surgical simulation, a simulator design that is easily created and portable for dissemination to neurosurgical trainees does not exist. At our institution, we have implemented the use of CAD design and 3DP to construct the SpineBox simulator, which is cost-effective, disposable, and able to replicate the cortico-cancellous interface for pedicle screw placement while providing a surgical environment that enables trainees to operate in realistic conditions with respect to instrument depth.

3D printing has been made widely available with desktop FDM 3DP models that are inexpensive and have a relatively small learning curve [24]. The common thermoplastics used for FDM printing materials have similar properties to cortical and cancellous bone and have been investigated to replicate human vertebrae for pedicle screw placement with excellent results [14,19,25]. Combining 3DP thermoplastics with dual-material printing or injection techniques are also distinct advantages of FDM printing for vertebral simulation compared with other 3DP methods [26]. Many teaching institutions have implemented the use of 3DP into their curriculum for anatomy and surgical education due to the relatively low cost and convenience of creating “in-house” models [20,25,27-29]. Despite these advantages, there are currently a limited number of open-access and freely available STL files for the surgical simulation that have been validated. This is partly due to industrial motivation and the potential for commercialization of simulator designs, as well as limitations in the reproducibility of construction methods for the simulators themselves. Investigators have examined the use of 3DP vertebral models placed within a foam operative cavity to simulate pedicle screw placement for the thoracolumbar spine, with excellent results in face, content, and validation [22]. The constructed simulators have shown promise for providing a quality surgical experience for neurosurgical trainees learning to place pedicle screws. Other investigators have explored fiberglass texture cervical vertebral models created through a reverse molding technique with 3DP molds placed within polyvinyl alcohol derivatives to create a cervical laminectomy simulation, with excellent results for task training and objective skill assessment [21]. Now, more than ever, innovation in 3DP simulators such as these models must continue to be investigated and produced in order to assist with the ever-growing needs of the surgical community in training the next generation of surgeons.

The SpineBox is able to replicate several validated methods of creating the cortico-cancellous interface for pedicle screw placement and to teach haptic feedback of both proper placement and breaches during pedicle access. The individual vertebral models in the simulator are designed hollow in order to allow the user to create infill gradients with their chosen slicing software before printing, or to fill with porous foams that accurately replicate the material properties of cancellous bone. Both of these methods have been previously validated as accurate methods of simulating cortical and cancellous bone [12,13,17]. The space around the vertebral models within the housing box can also be easily filled with soft foam, cotton, silicone, or other materials in order to simulate soft tissue structures that can impede visualization during screw placement, and allows the use of retractors during the simulation. The spinal canal of the vertebral models is also patent and can be filled with a Penrose drain, latex tubing, or other methods to reproduce dura and/or cerebrospinal fluid if a laminectomy simulation is desired as well.

Although this particular investigation was limited to the lumbar spine for demonstration of feasibility, our lab has created SpineBox units for cervical and thoracic levels as well, both for the practice of lateral mass and pedicle screw placement. Both of these CAD files are freely available for download and can be combined with foam in the same manner as the lumbar SpineBox model to create an in-house spine surgical simulator for teaching institutions worldwide (The SpineBox STL file can be downloaded for free at the Autodesk Online Gallery using the following link: https://bit.ly/39r2BLg).

## Conclusions

Neurosurgical education and simulation in the modern era must be able to reach beyond the bounds of physical confines. The recent world crisis caused by the COVID-19 pandemic has demonstrated the uncertainty surrounding the educational programs for neurosurgical trainees and exposed a widespread need for mobile and reproducible task-trainers that are low-cost and can still deliver a quality educational experience. The SpineBox is a freely available and easily 3D-printed simulator that allows for versatility in creating a quality educational tool for lumbar spine anatomy and pedicle screw placement using previously validated techniques for replicating the cortico-cancellous interface. By the open-access dissemination of this design, our team hopes to provide institutions across the world with a cost-effective means of spine surgical simulation for neurosurgical trainees and to encourage other rapid prototyping laboratories to investigate innovative means of creating educational surgical platforms in the modern era.
